# Mechanochemical green synthesis of hyper-crosslinked cyclodextrin polymers

**DOI:** 10.3762/bjoc.16.127

**Published:** 2020-06-29

**Authors:** Alberto Rubin Pedrazzo, Fabrizio Caldera, Marco Zanetti, Silvia Lucia Appleton, Nilesh Kumar Dhakar, Francesco Trotta

**Affiliations:** 1 Dipartimento di Chimica, Università degli Studi di Torino, Via Giuria 7, Torino 10125, Italy

**Keywords:** β-cyclodextrin, ball-milling, crosslinking, green chemistry, mechanochemistry, nanosponges

## Abstract

Cyclodextrin nanosponges (CD-NS) are nanostructured crosslinked polymers made up of cyclodextrins. The reactive hydroxy groups of CDs allow them to act as multifunctional monomers capable of crosslinking to bi- or multifunctional chemicals. The most common NS synthetic pathway consists in dissolving the chosen CD and an appropriate crosslinker in organic polar aprotic liquids (e.g., *N*,*N*-dimethylformamide or dimethyl sulfoxide), which affect the final result, especially for potential biomedical applications. This article describes a new, green synthetic pathway through mechanochemistry, in particular via ball milling and using 1,1-carbonyldiimidazole as the crosslinker. The polymer obtained exhibited the same characteristics as a CD-based carbonate NS synthesized in a solvent. Moreover, after the synthesis, the polymer was easily functionalized through the reaction of the nucleophilic carboxylic group with three different organic dyes (fluorescein, methyl red, and rhodamine B) and the still reactive imidazoyl carbonyl group of the NS.

## Introduction

The research in the fields of nanomedicine and nanotechnology has nowadays become predominant. Polysaccharides and, among them, starch derivatives such as cyclodextrins (CD), have recently emerged as they are safe, of low cost and biodegradable. Cyclodextrin nanosponges (CD-NS) are crosslinked cyclodextrin polymers characterized by a nanosized three-dimensional network. The reactive hydroxy groups of CDs allow them to act as polyfunctional monomers, permitting the crosslinking with bi- or multifunctional chemicals, such as dianhydrides, diisocyanates, diepoxides, and dicarboxylic acids, etc. The polarity and size of the polymer network can be easily tuned by varying the type of the crosslinker and degree of crosslinking, thus influencing the final properties [1–2].

Among the various bifunctional compounds that could be used as crosslinking agents, active carbonyl compounds, such as carbonyldiimidazole (CDI) and diphenyl carbonate have given interesting results in the last 20 years. The produced CD nanosponges, after crosslinking, comprise carbonate bonds between the cyclodextrin monomers. If this reaction is carried out under classical conditions in a *N*,*N*-dimethylformamide (DMF) solution, an amorphous crosslinked insoluble polymer is obtained. In general, carbonate nanosponges are insoluble in water and organic solvents and, unlike other types of NS, do not swell appreciably. In addition, they are nontoxic [1,3], stable up to 300 °C, and their structure allows modification through different functional groups before and/or after the synthesis. Carbonate nanosponges demonstrated promising results in removing organic compounds from wastewater and could therefore be suited for purifying water contaminated by persistent organic pollutants (POPs), such as chlorobenzenes and chlorotoluenes [2]. Moreover, CD nanosponges have found many applications in the pharmaceutical field, as for example, drug-delivery systems [3–5]: together with their capability of hosting drugs, they are biocompatible and nontoxic. In the last few years nanosponges were employed to encapsulate and release a wide variety of drugs [6–7], associated with an improvement in bioavailability and release kinetics. Nanosponges, as a powder, could be used as excipients in tablets, capsules, suspensions and dispersions, and topical formulations [8].

The most common NS synthetic pathway consists in dissolving the chosen CD in a suitable solvent, under continuous stirring, and then adding the crosslinker followed by a catalyst, if necessary. The solvents of choice were usually organic polar aprotic liquids, for example, *N*,*N*-dimethylformamide or dimethyl sulfoxide (DMSO). An alternative synthetic route relied on interfacial polymerization, where two immiscible solutions, one consisting of CDs dissolved in an alkaline aqueous solution and the other one containing the crosslinker in a chlorinated immiscible solvent, were mixed and stirred. At the interface between the solutions, crosslinking occurred [9].

In both cases the preparation of nanosponges, especially carbonate nanosponges, required the use of organic and often toxic solvents. The presence of these solvents affected the whole synthesis as the final material required rigorous purification extraction procedures with excess of water or volatile solvents to remove residual solvent inside the material’s structure. The removal of any synthetic contamination is essential for the use of nanosponges in the biomedical field. In addition, these synthetic procedures may also not be convenient for a possible future scale up of the reaction, as huge amounts of solvent have to be disposed of. Moreover, organic solvents are expensive and DMSO and DMF are difficult to recycle because of their high boiling points.

According to the Green Chemistry Principles, published in 1998 [10], processes have to be designed in order to “minimize the quantity of final waste and to avoid hazardous or toxic solvents”. Nanosponges themselves, nevertheless, are synthesized from starch derivatives and are biodegradable, so they are a very promising material from this point of view.

In this article a new, green synthesis of nanosponges through a mechanochemical approach is proposed.

Mechanochemistry relies on the application of mechanical forces (such as compression, shear, or friction) to drive and control chemical reactions, for example, using grinding or milling to transfer energy to chemical bonds [11]. Mechanochemical transformations are well established in inorganic chemistry and they are easily transferable to an industrial scale [12–13]. Mechanochemistry in organic chemistry, applied to organic syntheses and polymers has gained growing interest in recent years [14–18]. Mechanochemical syntheses are safe and represent efficient activation methods for greener processes, avoiding the use of solvents and reducing energy consumption. Recently, many examples for modifications of starch using ball milling have been reported. These included esterifications and etherifications of starch [19] and cellulose [20]. In 2017, Jicsinszky et al. obtained CD derivatives through a solid-state reaction, using a planetary ball mill [21–22] and by other green processes [23]. The main goal of this work was to obtain cyclodextrin nanosponges via a ball-milling-driven synthesis, having the same physicochemical characteristics as the material synthesized through the solvent-based approach.

Among the various types of cyclodextrin nanosponges, we chose carbonate NS, synthesized with 1,1-carbonyldiimidazole as the crosslinker, for the following reasons: the reaction was usually performed at 90 °C, therefore, the heating of the system related to the ball friction is not only acceptable but also useful for the kinetics of the reaction, and the solvent for solubilizing the reactants was DMF.

We herein present a new green synthesis of biodegradable polymers through a solvent-free procedure, displaying high potential of applications in various fields. After the synthesis, a significant amount of still reactive imidazoyl carbonyl groups within the NS structure were detected. These could be easily removed by washing with water at 40 °C leading to carbonate NS. On the other hand, the presence of a “tunable” quantity of remaining reactive groups could be used for further functionalization. In the present work this was demonstrated by a straightforward covalent coupling of the synthesized cyclodextrin nanosponges with selected organic dyes that are used as probe molecules with different structures (methyl red, rhodamine B, and fluorescein). The simple functionalization of the cyclodextrin NS, in this case via reactive imidazole moieties, is particularly interesting for a variety of applications. For instance, dye-modified cyclodextrins and CD derivatives have found wide use for the preparation of chemical sensors [24–25]. As CDs and consequently CD nanosponges can be easily coupled with fluorophores, they could find applications in the pharmacological area, for example, as biological markers, in image-guided therapies [26–28], and in conjugated drug delivery. This simple procedure also enables other active molecules to be grafted on the NS, to obtain conjugated nanocarriers, with reducing or in certain cases eliminating the need to use organic solvents.

## Results and Discussion

We performed various syntheses using different cyclodextrins with varying molar ratios of the cyclodextrin and crosslinker. Details of all polymers synthetized are collected in Table 1. The abbreviation βNS-CDI bm refers to a crosslinked β-cyclodextrin-based polymer (NS), obtained by crosslinking with CDI in a ball mill (bm). The same abbreviation was used for α and γ-cyclodextrins. The number following the crosslinker in the abbreviation refers to the molar ratio between the cyclodextrin and the crosslinker.

**Table 1:** Elemental analysis of NS-CDI polymers synthesized in a ball mill, after PSE and 2, 4 and 8 h in water at 40 °C. The presence of nitrogen was tested starting from *t*_0_ on plain NS after a simple wash with water and acetone at rt, after PSE extraction in acetone (high temperature and pressure, which efficiently removed imidazole, IMH), and after 2, 4 and 8 h of hydrolysis in water at 40 °C in order to remove the residual covalently bonded imidazolyl carbonyl group.

type of nanosponge	weight % of nitrogen		STD

αNS-CDI 1:4 bm	*t*_0_ (plain NS)	1.33	0.07
	after 2 h in H_2_O 40 °C	0.53	0.02
	after 4 h in H_2_O 40 °C	0.25	0.00
	after 8 h in H_2_O 40 °C	0.20	0.01
βNS-CDI 1:4 bm	*t*_0_ (plain NS)	2.69	0.12
	after 2 h in H_2_O 40 °C	0.63	0.01
	after 4 h in H_2_O 40 °C	0.31	0.01
	after 8 h in H_2_O 40 °C	0.23	0.02
γNS-CDI 1:4 bm	*t*_0_ (plain NS)	2.21	0.16
	after 2 h in H_2_O 40 °C	0.61	0.02
	after 4 h in H_2_O 40 °C	0.11	0.10
	after 8 h in H_2_O 40 °C	0.00	0.00
βNS-CDI 1:8 bm	*t*_0_ (plain NS)	6.39	0.05
	after 2 h in H_2_O 40 °C	2.56	0.03
	after 4 h in H_2_O 40 °C	1.31	0.03
	after 8 h in H_2_O 40 °C	0.40	0.03
βNS-CDI 1:2 bm	*t*_0_ (plain NS)	1.27	0.01
	after 2 h in H_2_O 40 °C	0.77	0.01
	after 4 h in H_2_O 40 °C	0.52	0.01
	after 8 h in H_2_O 40 °C	0.17	0.01

after pressurized solvent extraction			

βNS-CDI 1:2 bm	after PSE (acetone)	0.79	0.02
βNS-CDI 1:4 bm	after PSE (acetone)	1.19	0.07
βNS-CDI 1:8 bm	after PSE (acetone)	3.28	0.09

Three different ratios (1:2, 1:4, and 1:8) were tested using β-cyclodextrin. As usual for mechanochemistry [21,29], the synthetic procedure was easy to carry out and gave a high yield (>90%). Good mass balances (68%) were also achieved. The yield was calculated by considering the weight of the dried polymer with respect to the theoretical weight, equal to the sum of β-CD and C=O bridge between β-CDs. The solubility in various common solvents (water, acetone, ethanol, *N*,*N*-dimethylformamide or dimethyl sulfoxide, diethyl ether, and petroleum ether) of the new nanosponges was tested.

**Figure 1:**
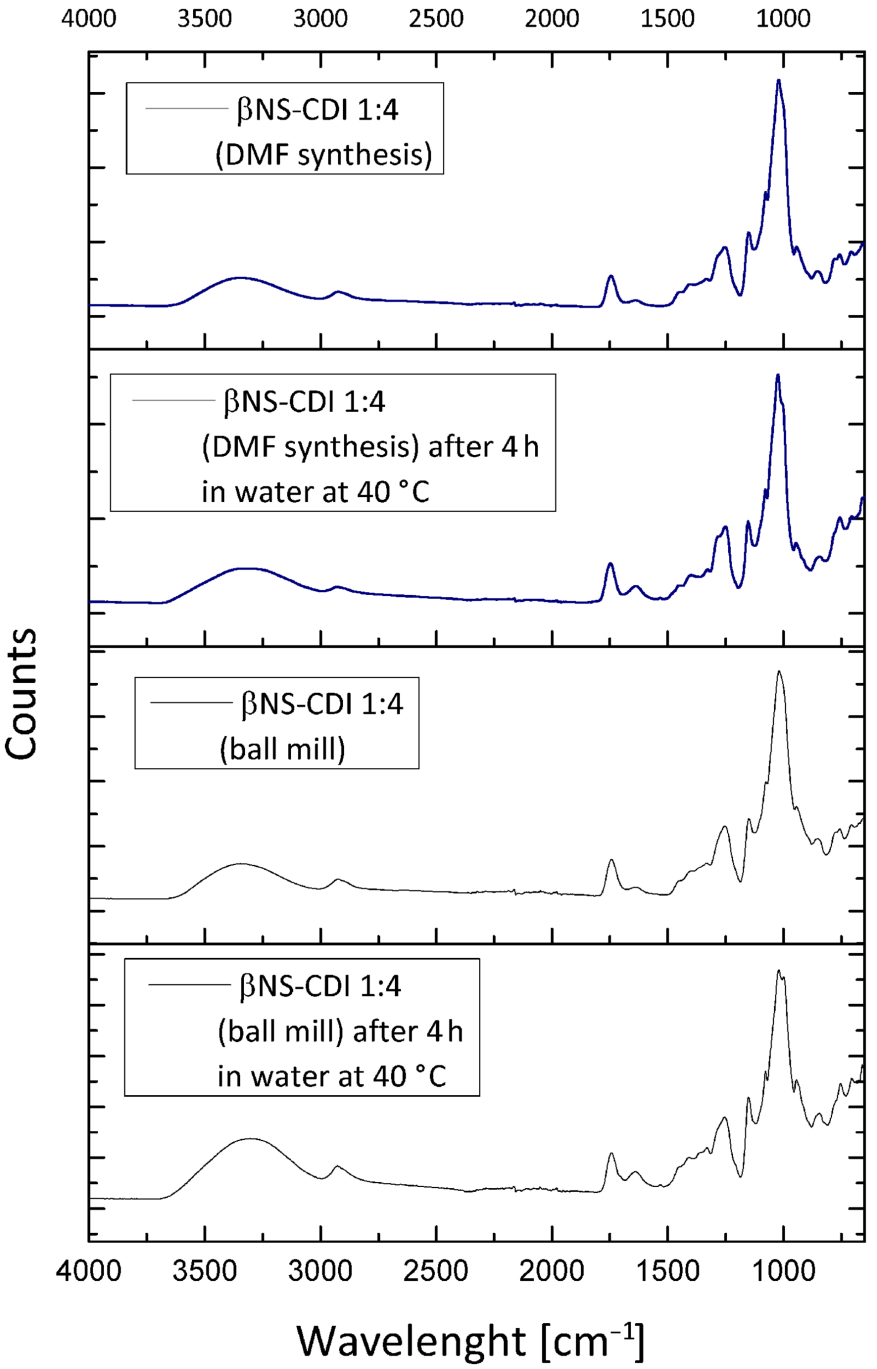
FTIR analysis of βNS-CDI 1:4, before and after treatment for 4 h in H_2_O at 40 °C, synthesized with and without solvent. The band of interest at around 1750 cm^−1^ assignable to the carbonyl group of the carbonate bond, was visible in all samples, even after treating for hours in H_2_O at 40 °C.

Like nanosponges obtained from batch experiments, also the ones from ball-mill synthesis, as expected, were insoluble in the tested solvents, in accordance with the formation of a crosslinked network and with data from previous literature [9]. Figure 1 reports FTIR spectra of the cyclodextrin NS, with a comparison between βNS-CDI 1:4 obtained through ball-mill synthesis and βNS-CDI 1:4 from synthesis in DMF and a comparison of the FTIR spectra after 4 h treatment in water at 40 °C. The FTIR spectra of βNS-CDI 1:4 obtained through different synthetic approaches, with and without solvent, exhibited a band at around 1750 cm^−1^ due to the carbonyl group of the carbonate bond. Even after hours of treatment in H_2_O at 40 °C the band was still present confirming the stability of the system. The spectra were almost superimposable, confirming the structure and the formation of carbonate bonds.

**Figure 2:**
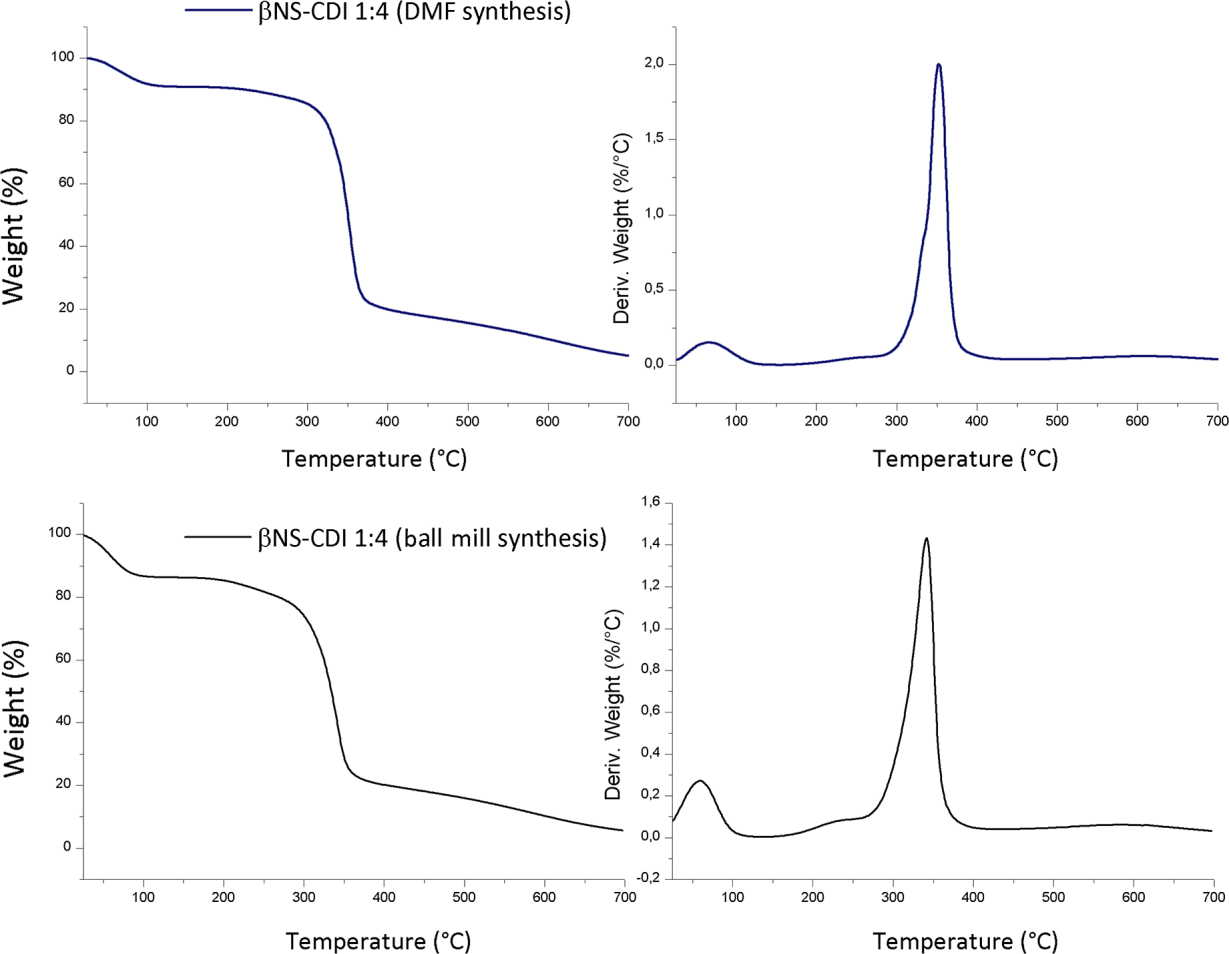
Thermogravimetric analysis of β-CD-based carbonate nanosponges, obtained through solution (DMF) and mechanochemical (ball mill) synthesis. Conditions: nitrogen flow, ramp rate 10 °C/min, rt to 700 °C.

**Figure 3:**
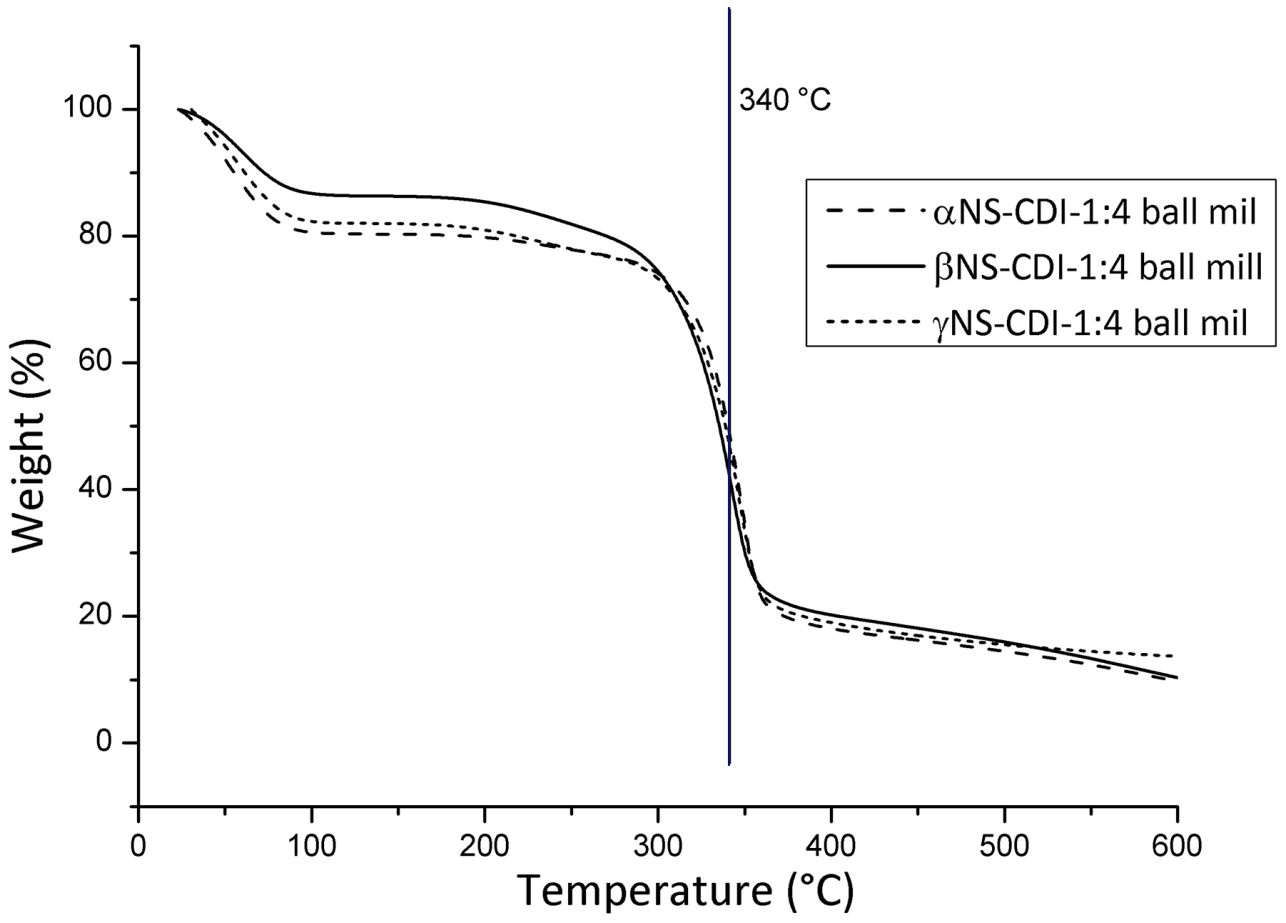
Thermogravimetric analysis of α, β and γ-CD-based carbonate nanosponges, obtained through ball-mill synthesis. Conditions: nitrogen flow, ramp rate 10 °C/min.

It was evident from the TG mass-loss curves and the corresponding derivate curves of the β-CD-based carbonate nanosponges (Figure 2), used for all further characterizations, that the crosslinked polymers synthesized by both methods exhibited a very close degradation path and, consequently, the same molecular structure was expected. The largest mass loss started above 300 °C for both βNS and the relative maximum rate peak was located at around 345 °C for both the βNS-CDI 1:4 from DMF and for the βNS-CDI 1:4 from ball mill. The initial mass loss present in both βNS-CDI 1:4 was due to adsorbed environmental water, always present when dealing with hygroscopic cyclodextrin-based nanoparticles. In addition to this, the particle size played a key role: the smaller the particles were the more the extended surface was exposed to the environmental humidity. In Figure 3, a direct comparison of the thermograms of αNS, βNS, and γNS synthesized through the ball-mill approach are shown.

As can be seen from Figure 3, the degradation paths were similar, however, with an interesting difference in the initial loss of water, which was due to the different water affinity of the CDs, resulting in different hygroscopy of the final materials.

**Figure 4:**
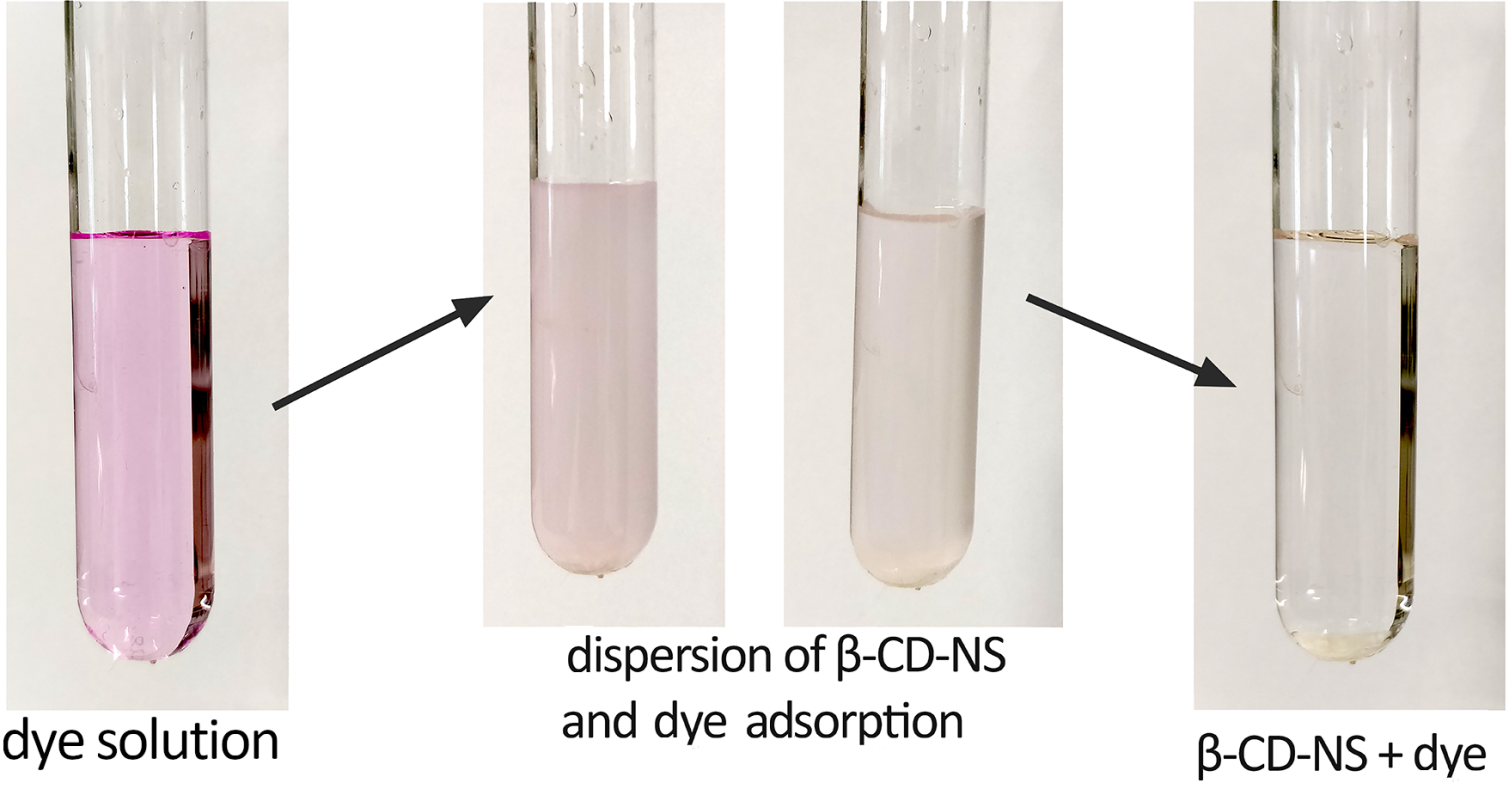
Adsorption of organic dyes by ball-mill synthesized β-CD-based carbonate nanosponges. Conditions: a small amount of NS (50 mg) was added to a clear solution containing the dye (10 mL). After the adsorption and the deposition of the NSs powder containing the organic dye, a clear solution was obtained.

In Figure 4, a test on the ability of the β-CD-based carbonate nanosponge obtained through ball-mill synthesis to remove organic compounds from aqueous solutions is shown. As can be seen methyl red was completely removed from its solution by adding a small amount of the bm carbonate nanosponge (50 mg for 10 mL of an aqueous solution of methyl red, 50 ppm).

As determined by DLS, the particle sizes of the βNS-CDI obtained through ball milling, were less than a micron (800–900 nm) immediately after the synthesis. Moreover, stable suspensions (also in time) with a particle size of around 200 nm for all βNS-CDI were obtained after a short cycle of ball milling with smaller spheres (for details, see Supporting Information File 1).

**Figure 5:**
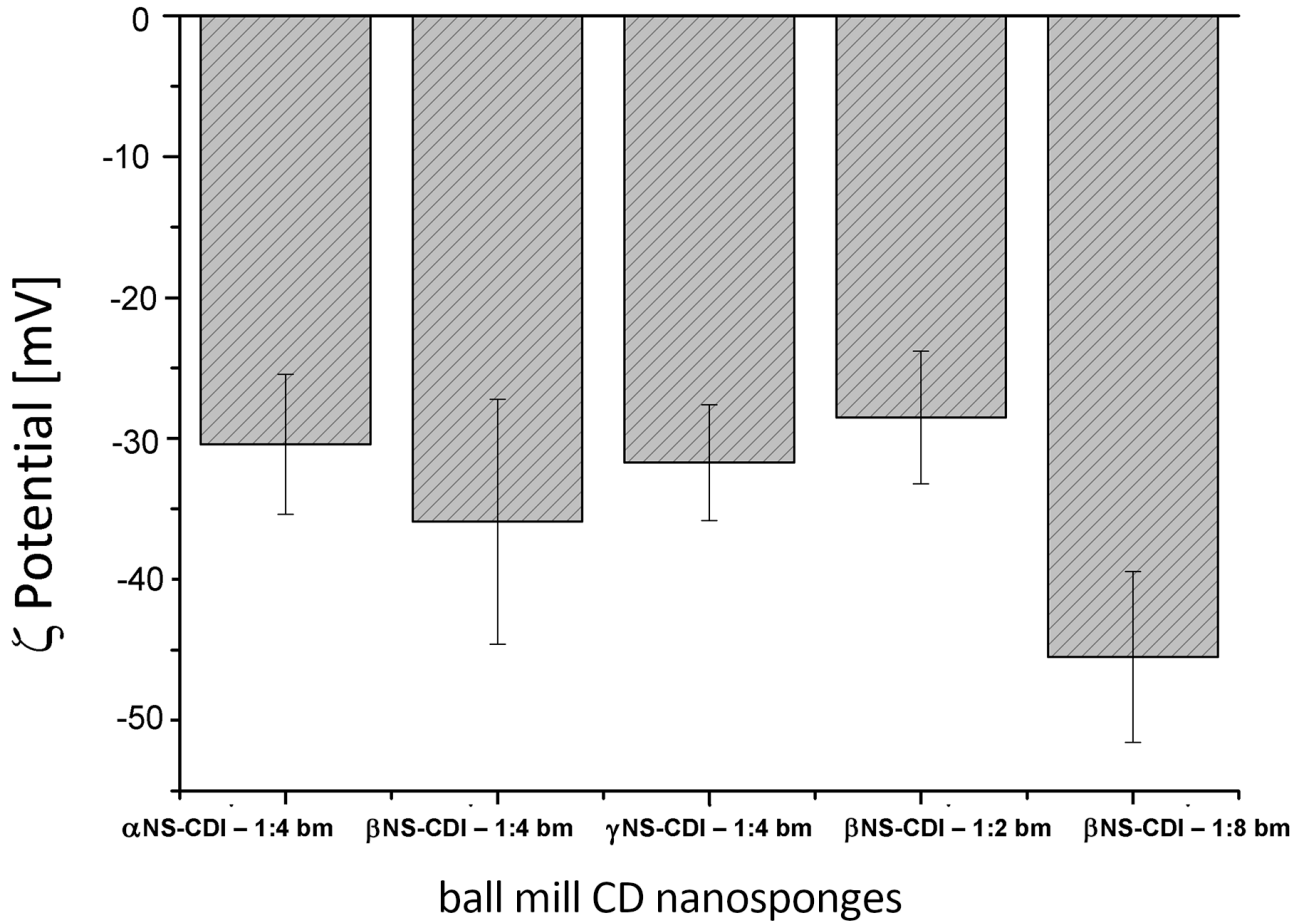
ζ-Potential of bm cyclodextrin nanosponges with relative STDev (mV).

The zeta-potential, Figure 5, of colloidal suspensions of bm βNS-CDI was tested for all of the nanomaterials. In general, the stronger the charge, the better the colloidal stability of the particles: βNS-CDI synthesized by ball-milling showed an interesting negative ζ-potential, which explained the stability of the dispersion.

As shown in Figure 5, all CD nanosponges exhibited a negative ζ-potential and this was in line with previous literature [2,9,30]. The negative charge seemed to be related to the amount of crosslinker: the larger the amount, the more negative the ζ-potential detected. The elemental analyses showed the presence of nitrogen even after pressurized solvent extraction (PSE) and this was attributed to the presence of reactive imidazolyl carbonyl groups (IM). In an ideal reaction, the carbonyl diimidazole should react completely with two hydroxy groups of CDs, forming a carbonate bond between two monomers and therefore releasing two imidazole molecules that are soluble in water and could be removed after synthesis.

CDI, however, may react asymmetrically forming only one bond with cyclodextrin leaving one of the two moieties reactive. This is consistent with what was reported in the literature: the first activation of an alcohol by carbonyl imidazole showed faster kinetics than the second one, which needed longer reaction times and/or a higher temperature (from 60 °C to 80 °C) to obtain a significant yield [31–32].

**Figure 6:**
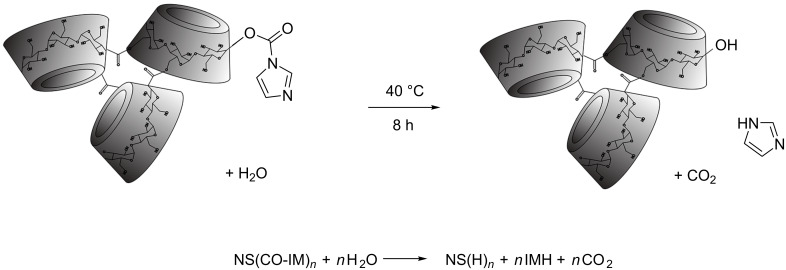
Hydrolysis of the imidazoyl carbonyl group in water at 40 °C.

**Figure 7:**
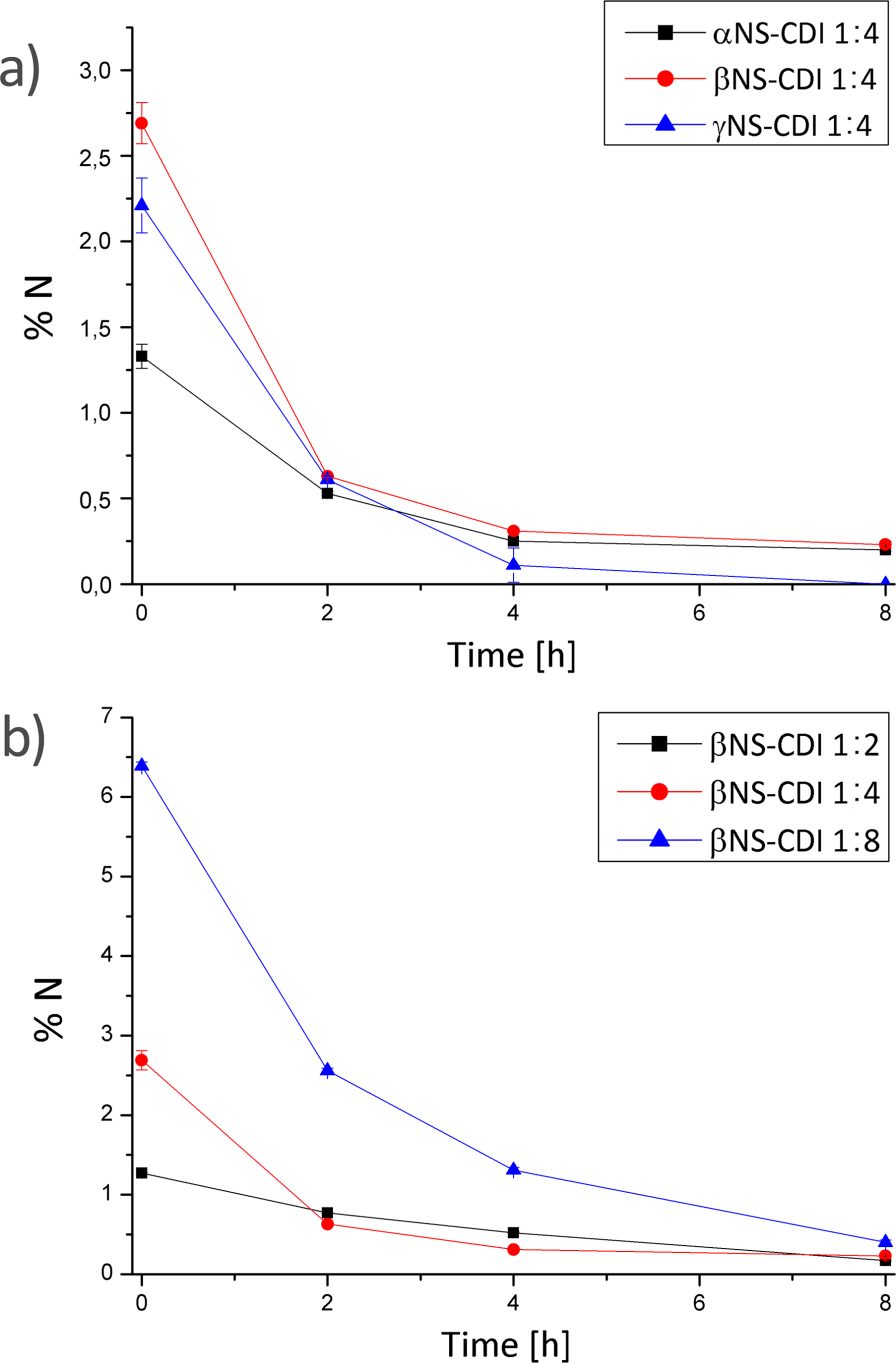
Nitrogen content in weight % in cyclodextrins NS-CDI from ball mill synthesis. a) comparison between different monomers with the same CD/CDI ratio and b) between the same CD crosslinked with different ratios.

To distinguish between the free imidazole (IMH) as byproduct and IM still able to form bonds (for example, with nucleophilic groups of active molecules) βNS-CDI was treated in two different ways: the first one entailed “hard” washing of the samples using acetone and PSE. Since acetone does not react by hydrolyzing the bond between the NS and the IM, the high pressure (120 bar) in PSE allowed the removal of the encapsulated IMH. The second treatment was longer and involved maintaining a small amount of material in water at 40 °C for 8 h, in order to effect hydrolysis (Figure 6):

Every 2 h, aliquots were withdrawn, washed with water to remove IMH, freeze-dried, and subjected to elemental analysis. The results were summarized in Table 1 and in Figure 7, nitrogen contents determined for different CD monomers reacted at the same CD/CDI ratio and for β-CD crosslinked with CDI at different ratios are shown. In Figure 1, the comparison between DMF and βNS-CDI 1:4 bm after 4 h treatment in water, confirmed what was stated in the previous paragraphs, such as the solubility and especially the consistency as far as the properties are concerned between the βNS-CDI obtained through the two different kinds of synthesis.

The results of the elemental analysis, reported in detail in Table 1 and Figure 7 agreed with what was expected from the reaction conditions and applied molar ratios: the βNS-CDI 1:8 exhibited the highest nitrogen content (around 6 wt %), before any further steps of purification. This result was consistent with the quantity of CDI involved in this nanosponge as it was 2–4 times higher as for the other βNS-CDI, assuming that the kinetics and reactivity were the same in all experiments. As a consequence, the IMH and unreacted IM contents were higher. Noteworthy was that most of the CDI reacted in the crosslinking step, as the amount of nitrogen after PSE using acetone dramatically decreased due to release of the IMH byproduct entrapped in the NS network. Furthermore, it could be stated that after about 8 hours, it was possible to eliminate almost completely both IMH and IM, using only water, from all nanomaterials.

The solubility and physicochemical properties of the NS-CDI were not depleted by these two different processes: all NS-CDI, with different molar ratios and different CDs, were still not soluble in any of the solvents tested previously (therefore, as shown in Figure 1, for βNS-CDI 1:4 the FTIR spectra were comparable). Hydrolysis, in fact, affected only the imidazolyl carbonyl groups and not the carbonate bond of the CD-NSs.

In order to confirm the presence of reactive imidazolyl carbonyl groups, which would make the functionalization of NS possible, an attempt was made on βNS-CDI 1:8 by using different organic dyes. First, βNS-CDI 1:8 was purified by PSE (and so, with only reactive IM left) and accurately milled. The functionalization, as described in the experimental section, was performed in DMSO, an organic solvent in which the material was insoluble but which was suitable for reactions in an anhydrous environment as in this case with organic dyes.

**Figure 8:**
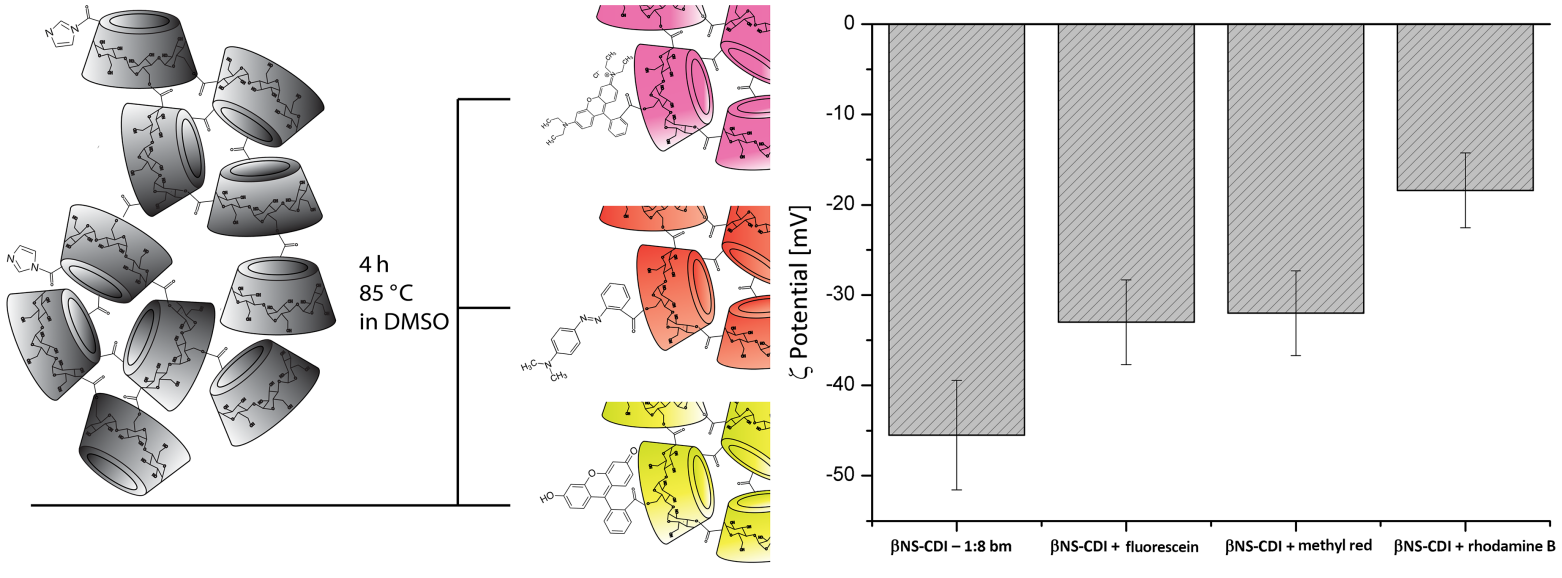
Simplified schematic reaction and procedure for obtaining the dye-functionalized βNS-CDI. Surface zeta potential of the plain and functionalized βNS-CDI 1:8.

The choice fell on three common, well known and widely investigated organic dyes, i.e., fluorescein, methyl red, and rhodamine B. They have a slightly different structures (and color), and also different surface charges but share a reactive nucleophilic carboxylic group. The simplified schematic reaction (previously reported by Staab [31] and more recently by Jadhav et al. [33]), with the relative ζ-pot of nanoparticles after functionalization, is presented in Figure 8. Through a simple reaction in closed vials with an excess (dye/CD ratio) of the organic dye, a covalent bond formation with the still reactive NS was achieved. As shown by elemental analysis, after PS extraction, the amount of nitrogen and therefore of reactive IM was very low. The experiment was conducted on nanosponges having 1:2, 1:4, 1:8 βCD/CDI ratios, and good results were obtained only with βNS-CDI 1:8. Even in case of βNS-CDI 1:8, if treated for 8 h in H_2_O at 40 °C (0.40% N), presented a low amount (≈0) of reactive IM, therefore the reaction did not occur at all, leading only to an inclusion complex with the dyes, which could be easily removed through a PS extraction with acetone). Moreover, this could be also explained since not all the reactive groups are freely accessible within the NS structure.

Carbonyl diimidazole can react with carboxylic acids at room temperature in many aprotic solvents (such as tetrahydrofuran, DMSO, or DMF) to form imidazolides in nearly quantitative yields, with the release of carbon dioxide and formation of IMH. The reaction kinetics of alcohols with *N*,*N*'-carbonyldiimidazole were generally slower, so the presence of reactive IM moieties may be particularly interesting for an easy functionalization via reaction with nucleophilic carboxy groups [31].

Additionally, the reaction with fluorescein was tested on both βNS-CDI 1:4 and βNS-CDI 1:2, which exhibited a considerably lower amount of nitrogen and consequently of reactive IM (nitrogen content: 0.79 wt % and 1.19 wt % for βNS-CDI 1:2 and 1:4, respectively, and 3.28 wt % for βNS-CDI 1:8 based on elemental analysis; see Table 1 for details). No appreciable differences in color or in ζ potential were observed. The presence of reactive imidazole was therefore crucial for this type of easy functionalization under mild conditions. The βNS-CDI 1:8 samples after reaction with the organic dyes and after two extractions with PSE using acetone, were colored. The functionalized materials were still insoluble in the tested solvents with no release of the dyes in any of them.

Of particular interest was the difference in the surface ζ-potential. It ranged from a very negative ζ-potential of around −45 mV (due to carbonate bonds and IM groups) to −33 mV, −32 mV, and −18,4 mV, after reaction with fluorescein, methyl red, and rhodamine B, respectively. The variation of the ζ-potential was consistent with the rhodamine B structure, exhibiting a positive charge. The elemental analyses of the samples containing rhodamine B, confirmed the presence of the organic compound within the structure (≈1 wt % N).

## Conclusion

Crosslinked cyclodextrin polymers, also called nanosponges (NS), were prepared via a new synthetic route based on mechanochemistry. The green synthetic route proposed here afforded a biodegradable polymer, displaying the same characteristics as cyclodextrin-based polymers synthesized in a solvent-based approach. The CD-based carbonate NSs were synthesized using an active carbonyl compound as a crosslinker, 1,1-carbonyldiimidazole, leading to an insoluble crosslinked polymer after 3 hours of ball milling. The synthesis was carried out using different cyclodextrins (α, β, and γ) and adopting the following molar ratios between cyclodextrin and the crosslinker, 1:2, 1:4 and 1:8.

The polymer obtained using the ball-mill method exhibited the same characteristics as a CD-based carbonate NS synthesized in a solvent and displayed insolubility in water and organic solvents. FTIR and TG analyses were performed on the new material, confirming the structure with a carbonate bond.

Nanoparticles, after cycles of ball milling and high-pressure homogenization had a mean diameter of less than 200 nm, as determined by DLS, and exhibited a negative ζ-potential (the most negative being at around −45 mV, measured for βNS at a 1:8 ratio of β-cyclodextrin/carbonyldiimidazole). Elemental analyses were conducted on all synthesized nanosponges in order to detect the presence of nitrogen derived from reactive imidazole moieties originating, in turn, from carbonyl diimidazole. This was confirmed by the reaction between the nanoparticles obtained and the nucleophilic carboxylic group of three different organic dyes, fluorescein, methyl red, and rhodamine B, leading to a colored (even after a PS extraction) functionalized material, with a less negative ζ potential.

## Experimental

### Materials

β-Cyclodextrin (β-CD), α-cyclodextrin (α-CD), and γ-cyclodextrin (γ-CD) were kindly provided by Roquette Italia SpA and Wacker Chemie. Carbonyldiimidazole (CDI, ≥97.0% (T)), 1,4-diazabicyclo[2.2.2]octane (DABCO, ReagentPlus^®^ grade, ≥99%), methyl red, rhodamine B, and fluorescein (for all dyes, declared dye content 95%), *N*,*N*-dimethylformamide (DMF, anhydrous, 99.8%), acetone (ACS reagent, ≥99.5%), and ethanol (ACS reagent, 96%) were purchased from Sigma-Aldrich (Munich, Germany) and used without further purification. The cyclodextrins were dried before use in an oven at 100 °C until constant weight. Elemental analyses were performed on a Thermo Scientific FlashEA 1112, using vanadium pentoxide purchased from Sigma. As planetary ball mill, a Retsch PM200 High Speed Planetary Ball Mill was used, with 20 sintered zirconium oxide balls of 10 mm diameter in 2 jars of 50 mL (10 balls per jar), also made from zirconium oxide. Rotation speed: sun wheel speed 600 rpm, changing rotation direction from clockwise to anticlockwise every 15 min. Thermogravimetric analyses were carried out on a Hi-res TGA 2050 Thermogravimetric Analyzer from TA Instruments. Parameters for all TG analyses were as the following: nitrogen flow, ramp rate 10 °C/min, rt to 700 °C. IR spectra of dried powders were recorded on a PerkinElmer Spectrum 100 FT-IR Spectrometer with 16 scans. Zeta potential and DLS measurements were performed on Zetasizer Nano ZS from Malvern Panalytical. All measurements were performed in triplicate. Solvent extraction for purifying samples was carried out with a pressurized solvent extractor (PSE) SpeedExtractor E-914 from Büchi.

#### Solvent synthesis of cyclodextrin nanosponges

The synthesis of α, β, γ carbonate nanosponges in a solvent is widely described in the literature [2–3]. The CDI crosslinked nanosponges synthesized for comparison with the ball-mill NS were prepared at different molar ratios of the respective anhydrous cyclodextrin and carbonyl diimidazole. For the 1:4 molar ratio, for example, the procedure was as follows: 3.00 g of α-cyclodextrin, 3.33 g of β-cyclodextrins and 4.05 g of γ-cyclodextrins (3.30 mmol) were dissolved in 10 mL of DMF (in three different round-bottomed flasks). After complete dissolution of the cyclodextrins, 2.00 g (13 mmol) of CDI as cross linker were added to each batch. The three flasks were heated at 90 °C for 3 h under stirring in an oil bath. Once the reaction was complete, the solid bulk obtained was crushed in a mortar, then extracted by PSE to remove the solvent, and unreacted crosslinker and CD. Finally, the polymers were ball-milled for 45 min.

#### Ball-mill synthesis of cyclodextrin nanosponges

The three CD crosslinked polymers were prepared using a ball mill in a one-step reaction without a solvent. The α, β, γ carbonate nanosponges were synthesized in the ball mill at 1:2, 1:4 and 1:8 molar ratios of the respective anhydrous cyclodextrin and carbonyldiimidazole. For example, for the 1:4 ratio synthesis, 3.38 g of α-cyclodextrin, 3.75 g of β-cyclodextrins and 4.56 g of γ-cyclodextrins were placed inside a 50 mL jar containing 10 zirconia balls. The amount of CDI added in each batch to maintain the 1:4 molar ratio was 2.25 g.

After 3 h of sun wheel rotation at 600 rpm, the reaction was completed and the external temperature was between 50–60 °C (the temperature according to previous studies reported in the literature was always indicated as lower than 72 °C under various conditions [22]). The finely ground powder was then dispersed in water and washed several times with deionized water and acetone. The samples were then extracted by pressurized solvent extraction (PSE), using acetone, to remove the residual imidazole in the NS structures.

#### Functionalization of cyclodextrin nanosponges

The functionalization was done following the same procedure for all samples: 500 mg of carbonate βNS 1:2, 1:4, 1:8 (molar ratio between β-CD and crosslinker, as mentioned previously) were dispersed in anhydrous DMSO. An excess of the organic dye (50 mg of dye, 10 wt % of the NS), methyl red, rhodamine B, and fluorescein, respectively, was then added to the dispersion, followed by heating at 85 °C in an oil bath for 4 h. The final product was washed with an excess of water and then extracted with acetone using PSE to remove the unreacted dyes.

## Supporting Information

File 1 Additional experimental data.
