# Supplementary material for: Mechanochemical green synthesis of hyper-crosslinked cyclodextrin polymers
Source: Beilstein J Org Chem. 2020 Jun 29;16:1554–63. doi: 10.3762/bjoc.16.127 (PMC7356557; doi:10.3762/bjoc.16.127)
Supplement: File 1 — Additional experimental data. [file Beilstein_J_Org_Chem-16-1554-s001.pdf]

BEILSTEIN JOURNAL OF ORGANIC CHEMISTRY

Supporting Information

for

Mechanochemical green synthesis of hyper-crosslinked cyclodextrin polymers

Alberto Rubin Pedrazzo, Fabrizio Caldera, Marco Zanetti, Silvia Lucia Appleton, Nilesh Kumar Dahkar and Francesco Trotta

Beilstein J. Org. Chem. **2020,** *16,* 1554–1563. doi:10.3762/bjoc.16.127

Additional experimental data

License and Terms: This is a supporting information file under the terms of the Creative Commons Attribution License (http://creativecommons.org/licenses/by/4.0). Please note that the reuse, redistribution and reproduction in particular requires that the authors and source are credited.
The license is subject to the *Beilstein Journal of Organic Chemistry* terms and conditions: (https://www.beilstein-journals.org/bjoc)

Size distribution

Size distribution of βNSCDI 1:8 from ball-mill synthesis, after cycle of wet ball milling, was determined by dynamic light scattering (DLS). Then the data from DLS analysis were crossed with data from nanoparticle tracking analysis (NTA), for a more accurate dimensional distribution. In *Figure S1*, the results are reported.

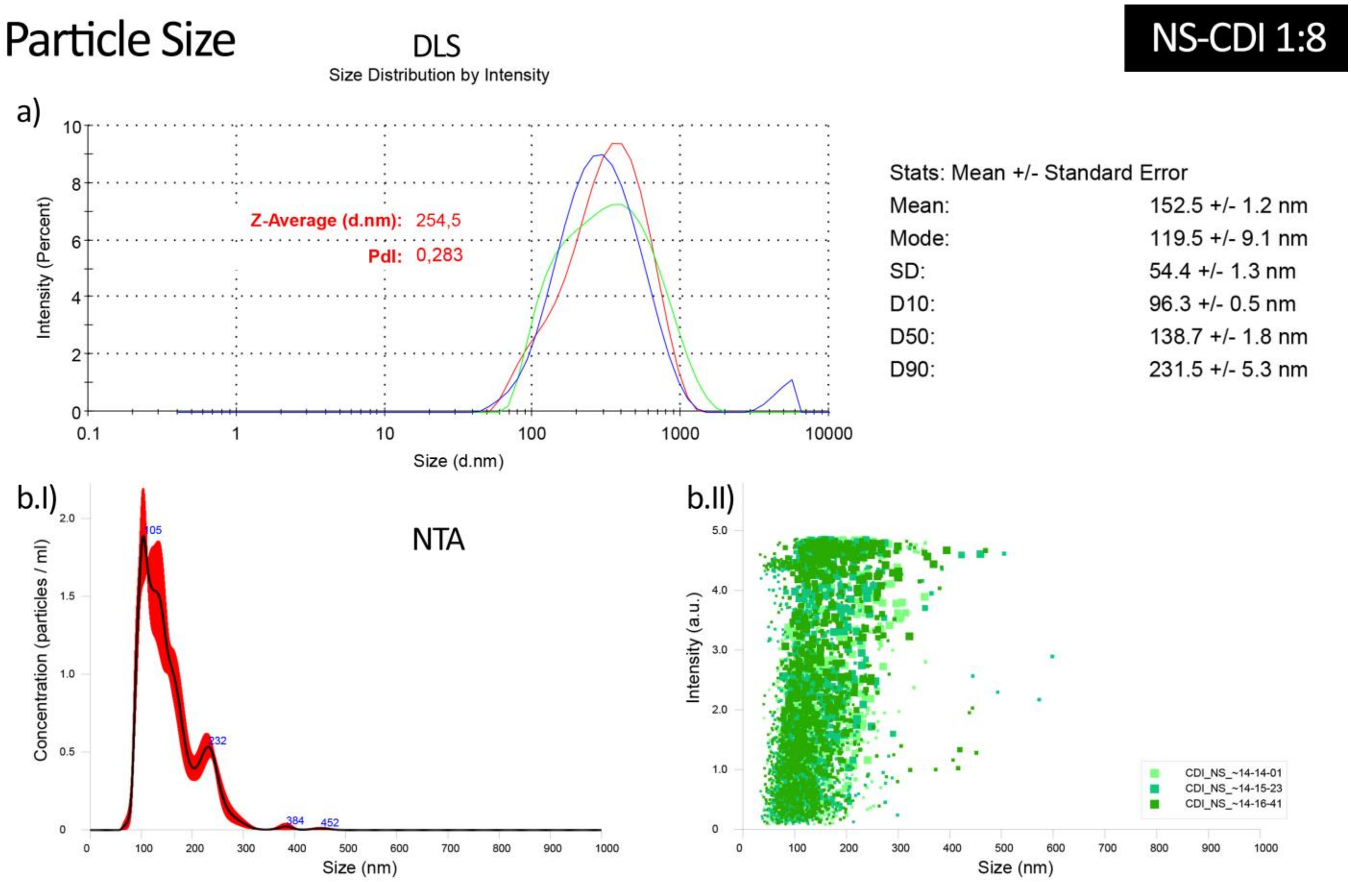

Figure S1: Size distribution of βNSCDI 1:8 from ball mill synthesis: a) DLS measurements b.I), b.II) distribution measurement via NTA.
